# “Real life” use of raltegravir during pregnancy in France: The Coferal-IMEA048 cohort study

**DOI:** 10.1371/journal.pone.0216010

**Published:** 2019-04-24

**Authors:** Pierre Gantner, Babacar Sylla, Laurence Morand-Joubert, Pierre Frange, Karine Lacombe, Marie-Aude Khuong, Claudine Duvivier, Odile Launay, Marina Karmochkine, Cédric Arvieux, Amélie Ménard, Lionel Piroth, Ana Canestri, Dominique Trias, Gilles Peytavin, Roland Landman, Jade Ghosn, on behalf of the Coferal-IMEA048 Study Group

**Affiliations:** 1 Hôpitaux Universitaires de Strasbourg, Laboratoire de Virologie, Strasbourg, France; 2 IMEA, CHU Bichat Claude Bernard, Paris, France Paris, France; 3 Sorbonne Universités, UPMC Univ Paris 06, INSERM, Institut Pierre Louis d'épidémiologie et de Santé Publique (IPLESP UMRS 1136), AP-HP, Laboratoire de Virologie, Hôpital Saint-Antoine, Paris, France; 4 APHP, Hopital Necker Enfants malades, Laboratoire de Microbiologie clinique, Paris, France; 5 EHU 7328, Institut Imagine, Université Paris Descartes, Paris, France; 6 Inserm UMR-S1136, IPLESP, AP-HP, Hôpital Saint Antoine, Department of Infectious Diseases, Paris, France; 7 Hôpital Delafontaine, Department of Infectious Diseases, Saint Denis, France; 8 APHP, Hopital Necker Enfants Malades, Department of Infectious Diseases, Centre d’Infectiologie Necker – Pasteur, IHU Imagine, Paris, France; 9 Université Paris Descartes, APHP, CIC Cochin Pasteur, Paris, France; 10 APHP, Hopital Européen Georges Pompidou, Department of Clinical Immunology, Paris, France; 11 COREVIH- Bretagne - CHU de Rennes, Rennes, France; 12 Institut hospitalo-universitaire (IHU) Méditerranée infection, Marseille, France; 13 Département d’Infectiologie, CHU Dijon, Dijon, France; 14 APHP, Hôpital Tenon, Maladies Infectieuses, Paris, France; 15 Merck Sharp Dohme, Paris, France; 16 APHP, Hopital Bichat Claude Bernard, Department of Pharmacology-Toxicology, Paris, France; 17 INSERM IAME UMR-S 1137, Université Paris Diderot, Paris, France; 18 APHP, Hopital Bichat Claude Bernard, Department of Infectious Diseases, Paris, France; Institut Hospital del Mar d’Investigacions Mediques, SPAIN

## Abstract

**Introduction:**

Limited “real life” data on raltegravir (RAL) use during pregnancy are available. Thus, we aimed at describing effectiveness and safety of RAL-based combined antiretroviral therapy (cART) in this setting.

**Methods:**

HIV-1-infected women receiving RAL during pregnancy between 2008 and 2014 in ten French centers were retrospectively analysed for: (1) proportion of women receiving RAL anytime during pregnancy who achieved a plasma HIV-RNA (pVL) < 50 copies/mL at delivery, and (2) description of demographics, immuno-virological parameters and safety in women and new-borns.

**Results:**

We included 94 women (median age, 33 years) of which 85% originated from Sub-Saharan Africa and 16% did not have regular health insurance coverage. Sixteen women were cART-naïve (median HIV diagnosis at 30 weeks of gestation), whereas 78 were already on cART before pregnancy (40% with pVL < 50 copies/mL). RAL was initiated before pregnancy (n = 33), during the second trimester (n = 11) and the third trimester of pregnancy (n = 50). No RAL discontinuations due to adverse events were observed. Overall, at the time of delivery, pVL was < 50 copies/mL in 70% and < 400 copies/mL in 84% of women. Specifically, pVL at delivery was < 50 copies/mL in 82%, 55% and 56% of cases when RAL was started before pregnancy, during the second or third trimester of pregnancy, respectively. Median term was 38 weeks of gestation, no defect was reported and all new-borns were HIV non-infected at Month 6.

**Conclusions:**

RAL appears safe and effective in this “real-life” study. No defect and no HIV transmission was reported in new-borns.

## Introduction

In addition to the large decrease in HIV/AIDS-related mortality and morbidity, another major and early success of combined antiretroviral therapy (cART) has been the dramatic decrease of HIV mother-to-child transmission (MTCT). Indeed, current MTCT rates globally fall below 5% [1], this risk reaching almost zero for women on successful cART before pregnancy and maintaining success until delivery [2]. However, despite major improvements in antiretroviral drugs, the composition of cART during pregnancy remains challenging [3]. No antiretroviral drug can be considered totally safe during pregnancy, and several severe adverse events have been reported in new-borns exposed to cART *in utero*, such as heart defects with zidovudine (ZDV) exposure [4], neonatal adrenal dysfunction with lopinavir/ritonavir exposure [5], discrepant results between preclinical and clinical studies about neurologic defects with efavirenz [4, 6, 7], which is now considered as safe as other cART [8], risk of cancer during childhood with didanosine exposure [9] and alteration of DNA repair and telomere maintenance genes with ZDV/tenofovir exposure [10]. Until recently, international and French guidelines recommended the use of two nucleoside reverse transcriptase inhibitors (NRTI), namely emtricitabine plus tenofovir disoproxil fumarate (TDF/FTC) or abacavir plus lamivudine (ABC/3TC) in combination with a ritonavir-boosted protease inhibitor (bPI), as preferred regimen during pregnancy [11–13]. The availability of integrase strand-transfer inhibitors (INSTI), together with some concerns about a potential association between the use of bPI and premature delivery [14–16], could be a “game changer” in the field of cART during pregnancy. Indeed, the ability of INSTI to rapidly control HIV replication is very appealing [17, 18], especially in late presenting women (i.e. women who arrive late in the pregnancy follow-up for HIV care). However, the use of dolutegravir in the periconceptional period, which has been associated with neural tube defects [19] and need to be further confirmed, has recently raised some concerns about INSTI usage during pregnancy. Conversely, no signal for birth defects in pre-clinical studies was associated with the use of raltegravir (RAL); the first available INSTI, that was neither mutagenic nor clastogenic in a series of *in vitro* and animal screening tests [20, 21]. In 2015, when the Antiretroviral Pregnancy Report had gathered data sufficient enough to rule out a two-fold increase in risk of overall birth defects, RAL has been included as a preferred agent in pregnancy according to the U.S. Department of Health and Human Services [22]. Furthermore, European AIDS Clinical Society also included RAL use during pregnancy as a recommended option since 2017 [11–13]. In this context, to assess RAL use during pregnancy is both safe and effective; we conducted a retrospective cohort analysis of HIV-infected women who received a RAL-based regimen during pregnancy in France.

## Patients and methods

### Study design and study population

We conducted a comprehensive retrospective chart review of all HIV-1-infected pregnant women, followed in ten selected clinical centers across France, who received a RAL (400 mg twice daily)-based cART for at least 15 days anytime during pregnancy, regardless of pregnancy outcome, between 2009 and end of 2014 (Coferal-IMEA048 cohort study). Inclusion period did not extend beyond 2014 because a national trial assessing the pharmacokinetics properties of RAL during the third trimester of pregnancy started in France as of 2015 (NCT02099474). Socio-demographic, virologic, immunologic and therapeutic characteristics of pregnant women were collected. The precise timing and the reason for initiation of RAL were also reported, as well as pregnancy outcome, neonates’ clinical characteristics and their HIV status at 6 months of age.

The primary endpoint was the proportion of plasma HIV-RNA (pVL) suppression close to (maximum 4 weeks before) or at the time of delivery. Secondary endpoints included delivery mode, the HIV status of new-borns from birth until month 6 of age, and safety parameters of the infant and the mother. Safety parameters of the infant included birth weight, gestational age, and birth defects. Maternal safety parameters included clinical and biological tolerance of RAL-based cART.

### Ethics

Retrospective oral non-opposition for their clinical data to be anonymized and then analyzed for research purposes was obtain from study participants. The Ethics Committee “Comité de protection des personnes Ile de France XI” approved the study.

### Statistical analysis

The proportion of pregnant women with pVL < 50 and < 400 copies/mL at delivery (main outcome measure) was reported as percentage. The proportion of women experiencing a pVL decay of more than 2 log_10_ copies/mL between the time of RAL initiation and delivery was calculated for women with a pVL > 5000 at baseline (i.e. RAL initiation). Fisher’s exact test was used to compare proportions of pVL < 50 copies/mL at delivery according to the timing of RAL initiation in R version 3.1.1 (R Foundation, Vienna, Austria).

## Results

### Characteristics at baseline

Ninety-four pregnancies in 94 HIV-1 infected women with a median age of 33 years were included in the present analysis (Table 1). Women primarily originated from Sub-Saharan Africa (85%) and 16% of them did not have regular health insurance coverage (none or State Medical Aid). At the time of RAL initiation, 16 women (17%) were cART-naïve and late presenters (i.e. women who arrive late in the pregnancy follow-up for HIV care), with an HIV diagnosis at a median of 30 weeks of gestation (range, 22–37), and 78 women (83%) were already on cART before pregnancy (40% of them with a pVL < 50 copies/ml) including 33 women on RAL. Among the 16 late presenters, RAL was used with two NRTI (n = 14) and/or one bPI (n = 13). Among the 78 cART-experienced women, RAL was associated with two NRTI (n = 70) and/or a bPI (n = 54) and/or a non-nucleoside reverse transcriptase inhibitor (n = 4) and/or a CCR5 antagonist (n = 3). For further details on cART-backbone please refer to Table 2.

**Table 1:** Baseline characteristics of the study participants.

Baseline characteristics	median (range) or n/total (%)
Age (years)	33 (20–45)
Mode of transmission	
Heterosexual	86 / 94 (92%)
Blood transfusion	1 / 94 (1%)
MTCT	2 / 94 (2%)
Unknown	5 / 94 (5%)
Geographical origin	
Sub-Saharan African	80 / 94 (85%)
Caucasian	8 / 94 (9%)
Maghrebin	4 / 94 (4%)
Asian	2 / 94 (2%)
Medical insurance coverage	
General social security coverage	58 / 94 (62%)
Universal Medical Coverage (CMU)	21 / 94 (22%)
State Medical Aid (AME)	12 / 94 (13%)
None	3 / 94 (3%)
HIV Co-infections	
HIV / HBV	5 / 94 (5%)
HIV / HCV	0 / 94 (0%)
First HIV-RNA during pregnancy (copies/mL)	
Women with HIV-RNA < 50 copies/mL (n)	38 / 94 (40%)
Women with HIV-RNA > 50 copies/mL (n)	56 / 94 (61%)
Detectable HIV-RNA (copies/mL)	9823 (63–638388)
First CD4 T-cell count during pregnancy (/mm^3^)	448 (4–986)
cART History	
Naïve	16 / 94 (17%)
Treatment-experienced	78 / 94 (83%)
Obstetrical history	
Number of previous pregnancy (n)	3 (1–8)
Previous twin or multiple pregnancy	2 / 94 (2%)

MTCT, Mother-To-Child Transmission; cART, combined Antiretroviral Therapy.

**Table 2:** Detailed RAL-based regimen backbones.

**cART-naïve women, n**		**16**
**NRTI**		14 (88%)
FTC/TDF	Emtricitabine/tenofovir disoproxil fumarate	8 (50%)
AZT/3TC	Zidovudine/lamivudine	6 (38%)
**bPI**		13 (81%)
LPV/r	Lopinavir/ritonavir	8 (50%)
DRV/r	Darunavir/ritonavir	4 (25%)
ATZ/r	Atazanavir/ritonavir	1 (6%)
**cART-experienced women, n**		**78**
**NRTI**		70 (90%)
FTC/TDF	Emtricitabine/tenofovir disoproxil fumarate	34 (44%)
ABC/3TC	Abacavir/lamivudine	14 (18%)
AZT/3TC	Zidovudine/lamivudine	7 (9%)
ABC	Abacavir	7 (9%)
TDF	Tenofovir disoproxil fumarate	5 (6%)
ZDV	Zidovudine	2 (3%)
3TC	Lamivudine	1 (1%)
**bPI**		54 (69%)
DRV/r	Darunavir/ritonavir	35 (45%)
ATZ/r	Atazanavir/ritonavir	8 (10%)
LPV/r	Lopinavir/ritonavir	9 (12%)
SQV/r	Saquinavir/ritonavir	1 (1%)
FPV/r	Fosamprenavir/ritonavir	1 (1%)
**NNRTI**		4 (5%)
ETR	Etranvirine	3 (4%)
NVP	Nevirapine	1 (1%)
**CCR5 antagonist**		3 (4%)
MVC	Maraviroc	3 (4%)

cART, combined Antiretroviral Therapy; NRTI, Nucleoside Reverse Transcriptase Inhibitor; NNRTI, Non-Nucleoside Reverse Transcriptase Inhibitor; bPI, boosted Protease Inhibitor.

Overall, RAL-based cART was initiated: (i) before pregnancy in 33 women, (ii) during the second trimester of pregnancy in 11 women (2 switches for clinical intolerance of bPI and 9 for cART intensification because of a pVL > 50 copies/mL), and (iii) during the third trimester of pregnancy in 50 women (34 for cART-intensification and 16 cART initiation in naïve late presenters pregnant women). According to the timing of RAL initiation, 22/33, 1/11 and 15/50 women had a pVL < 50 and copies/mL before pregnancy, during the second trimester, and during the third trimester, respectively. In women with a pVL > 50 copies/ml (80%), median pVL was 1740 copies/mL (range, 55–6 680 000) at RAL initiation.

### Effectiveness and safety for the mothers

**Fig 1:**
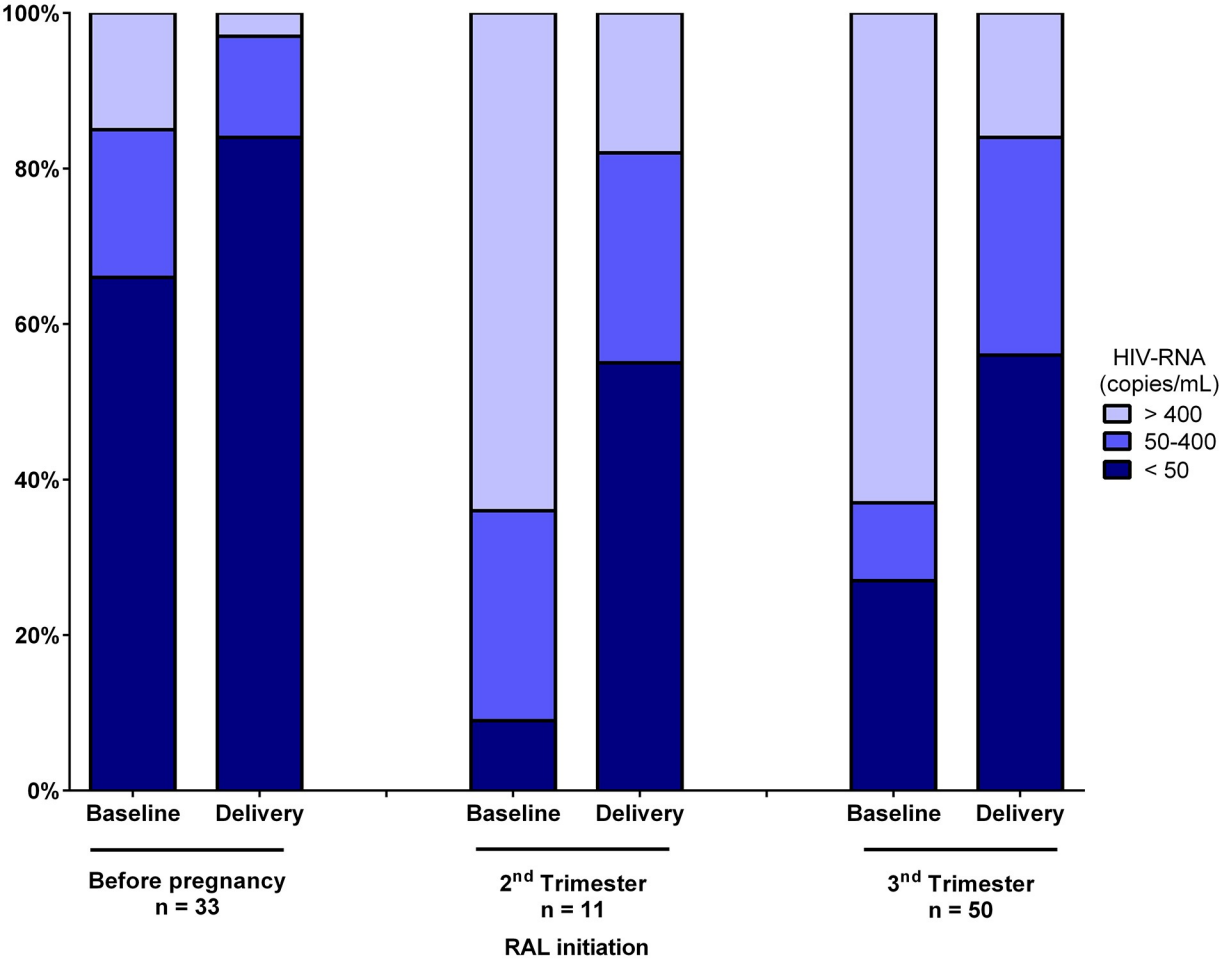
HIV-RNA range at baseline and delivery according to the timing of raltegravir initiation. RAL, raltegravir.

Overall, at the time of delivery, pVL was < 50 copies/mL in 70% and < 400 copies/mL in 84% of women, respectively. Specifically, pVL was < 50 copies/mL at delivery in 82%, 55% and 56% of women when RAL was started before pregnancy, during the second trimester or during the third trimester, respectively (Fig 1). RAL initiation before pregnancy was associated with significantly higher rates of virological control achievements when compared to initiations during the third trimester of pregnancy (Fisher’s exact test, p = 0.02). For women with a pVL > 50 copies/mL at delivery, median pVL was 185 copies/mL (range, 55–39068). Of note, among women with a pVL > 5000 copies/mL at RAL initiation (n = 36), a pVL decay greater than 2 log_10_ copies/mL between RAL initiation and the time of delivery was observed in 87% of them. Median CD4+ T-cell count at delivery was 560 /mm^3^ (range, 63–1698). RAL-based cART was well tolerated during pregnancy with no discontinuation due to adverse event. Of note, during labour, additional ZDV infusion was performed in 60% of women according to French guidelines [12].

### Birth outcomes

All the 94 pregnancies (92 singleton, 2 twins) led to live birth. Median delivery term was 38 weeks of gestation (range, 28–41), with vaginal delivery in 48% and caesarean section in 52% of cases. Of note, 18 (19%) of new-borns were premature (< 37 weeks of gestation). Median new-borns’ weight was 3.1 kg (range, 0.8–4.5) and no birth defect was reported. All of them received ZDV during the post-partum period [12], and were HIV non-infected by HIV-DNA/RNA measurements at Month 6 of age. We thus observed a transmission rate of 0% (95% CI, 0–6) in women with a pVL < 50 copies/mL at delivery and of 0% (95 CI, 0–12) in women with a pVL > 50 copies/mL at delivery.

## Discussion

Recommended cART options remain limited for HIV-1 pregnant women despite the development of new antiretroviral drugs over the past decade. We report here a comprehensive retrospective analysis of HIV-1 infected women receiving a RAL-based cART during preganncy. Overall, a pVL < 50 copies/mL at the time of delivery was achieved in more than two third of women initiating RAL, although 60% of them were not virologically-suppressed at RAL initiation. Of major interest, this “real-life” study provides data for specific populations usually excluded from clinical trials. In particular this analysis: (i) included women initiating a RAL-based regimen at different time points, including late presenters, (ii) is representative of socio-demographic characteristics of HIV-infected pregnant women epidemiology in France, thus including a high proportion of Sub-Saharan Africa migrants, and (iii) described data for women with no regular health care insurance, who therefore cannot participate in clinical trials.

To date, two prospective studies described efficacy of RAL-based cART in HIV-infected pregnant women. The multicenter study of Blonk *et al*., analyzed 22 women (a third of them had initiated RAL before pregnancy) for RAL efficacy, safety and pharmacokinetics during pregnancy and showed that 86% of them achieved pVL < 50 copies/mL at the time of delivery [21]. Recently, Puthanakit *et al*. showed that among women at high risk of MTCT initiating RAL at a gestational age ≥ 32 weeks (n = 154), 45% were undetectable at the time of delivery [23]. Of note, RAL was safe for both women and infants; however, 6 infants were HIV-infected in this trial (3 *in utero* and 3 peripartum transmissions). Besides, RAL effectiveness data in a clinical setting are scarce, and mostly limited by a small number of individuals. Nobrega *et al*. described a pVL < 50 copies/mL rate at delivery of 50% in 14 pregnant women starting RAL due to late presentation [24]. Moreover, 57% of HIV-infected women receiving RAL for intensification or as late presenters (n = 28) achieved a pVL < 50 copies/mL in an another retrospective single center study [25]. As expected, when compared to other cART regimens, INSTI-receiving women (n = 39) showed more rapid pVL decay than with other drugs (n = 62) in a retrospective multicenter study [26]. However, in a last single center retrospective study, no significant difference in achieving pVL suppression was observed between HIV-1 pregnant women receiving an INSTI (n = 7) an those receiving a bPI (n = 14) based cART, matched for baseline viremia [27]. Altogether, INSTI, and more specifically RAL effectiveness during pregnancy in terms of pVL suppression at delivery, ranged from 45% to 86%, mostly depending on: (i) baseline pVL, (ii) the timing of RAL introduction, and (iii) adherence. Our results, though obtained from a “real life” cohort, were in the higher range of effectiveness of these previous reports. Viral suppression rates were thus satisfactory when considering that most women of our cohort initiated RAL lately, during the second and the third trimester of pregnancy. Indeed, we observed rapid pVL decay on RAL in women with high levels of HIV replication at baseline.

As regards to safety in women and infants, the present study, in keeping with previous reports, demonstrated few tolerability issues and low rates of RAL discontinuations due to adverse events [21, 23–25]. Accordingly, RAL use in HIV-infected neonates is now recommended by WHO [28]. Interestingly, few previous studies included women receiving RAL-based cART since conception. As previously described by Blonk *et al*. in 7 new-borns exposed to RAL during all pregnancy [21], we did not report any birth defect in the 33 new-borns whose mothers started RAL before conception. These reassuring results are in line with the data of the Antiretroviral Pregnancy Registry, which showed no difference in overall risk of birth defects for RAL compared with background rate for major birth defects in the US reference population [22]. Moreover, no neonate was infected by HIV *in utero* or in the *peri-partum* period in our cohort. In May 2018, a warning about an unexpected high rate of neural tube defects in HIV-infected pregnant women who received a dolutegravir (DTG, an INSTI)-based regimen from the time of conception raised serious concerns with regards to the use of dolutegravir [19]. Such defects might not be related with the INSTI as a drug class given that no similar effect has been reported with RAL in the literature so far. Overall, our data further emphasize that the use of RAL during pregnancy is safe in addition to almost no drug-drug interactions.

Recently, a pharmacokinetic study enrolling 43 women showed that the standard dosage of RAL 400 mg twice daily yielded RAL plasma free concentrations during the third trimester of pregnancy only 16% lower than those measured during the post-partum period, thus confirming that the standard dosage of RAL 400 mg twice daily did not need any adjustment during the third trimester of pregnancy [29]. Conversely, decreased elvitegravir (EVG, an INSTI) blood plasma concentrations were reported during the second and the third trimester, which further hampers EVG use during pregnancy [30].

Our study has several limitations inherent to its retrospective design. Indeed, some medical records might be partly incomplete, however, the study population is representative of HIV-infected pregnant women in France. Moreover, it is the largest non-prospective study on RAL use during pregnancy so far. We also acknowledge that the population size might limit the description of rare birth defects. Another limitation to our study is the absence of a comparator group, which is complex to establish in a non-randomized clinical setting during pregnancy. Due to the retrospective and multicenter design of the study, and the fact that women are followed in both infectious diseases department and in the maternity wards, data on genotypic resistance tests and adherence assessments were not available for the present study.

Overall, we report that RAL use during pregnancy was effective and safe in a “real life” setting. Specifically, due to rapid pVL decay, more than two third of women tend to or achieved virological control on RAL-based cART at the time of delivery, which in turns leaded to the absence of MTCT. Conversely to other INSTI as EVG for pharmacokinetics reasons [30], or DTG for safety reasons [19], we suggest that RAL can be safely used during pregnancy, which is of particular interest in cART-naïve late presenters or as an intensification strategy, and that no birth defect was reported in women already receiving RAL before pregnancy (i.e. at the time of conception).
